# The effects of environmental enrichment on hatchery-performance, smolt migration and capture rates in landlocked Atlantic salmon

**DOI:** 10.1371/journal.pone.0260944

**Published:** 2021-12-02

**Authors:** Matti Janhunen, Jorma Piironen, Anssi Vainikka, Pekka Hyvärinen

**Affiliations:** 1 Natural Resources Institute Finland (Luke), Joensuu, Finland; 2 Department of Environmental and Biological Sciences, University of Eastern Finland, Joensuu, Finland; 3 Natural Resources Institute Finland (Luke), Paltamo, Finland; Universiteit Gent, BELGIUM

## Abstract

Enrichment of rearing environment with natural elements has been suggested to improve the welfare and post-release survival of cultured fish. We studied the combined effects of shelter structures, periodical water flow and water level changes on pre- and post-release performance of critically endangered landlocked Atlantic salmon (*Salmo salar* m. sebago). Relative to standard (plain) rearing tanks, provision of enrichment improved fish condition factor and survival during the first year of rearing when most mortality was attributable to parasitic and bacterial infections. The consequent higher density in enriched tanks probably induced greater growth variation and more dorsal fin damages than found in fish of standard tanks. Possibly this was partly due to the applied changes in water level. Experimentally determined smolt migration tendency at age 3 did not differ, on average, between the rearing groups, but enriched-reared fish showed clearly less variation in total movement activity than standard-reared fish. Experimental angling in earthen ponds did not suggest divergent vulnerability between the differentially reared fish at age 3, but decreased condition during the preceding growth season increased vulnerability to fishing. Based on long-term post-stocking tag returns in large-lake fisheries, fish length at release but not rearing method affected the capture rates of fish released at age 2. When released at age 3 the fish grown in enriched environment had a higher risk to be captured with stationary gears and earlier by hook and line gears compared to standard-reared conspecifics. Earlier time of maximal smolt migration activity was associated with an increased risk of being captured. We suggest that environmental enrichment may modulate growth- and behavior-related qualities that indirectly increased the vulnerability to fishing in natural conditions but not in experimental setting. The favorable effects of enrichment on early survival encourages adopting enriched rearing practices in supportive breeding of landlocked salmon.

## Introduction

Enormous quantities of hatchery-reared fish are being released to natural waters to support both fisheries and conservation of fish resources, but unfortunately, often without systematic evaluation of the success of the stockings [1]. Capture rates of stocked fish have been regularly compromised by their low post-release survival [2–4]. While a growing body of research indicates that captive rearing may bring about persistent (genetic) changes that negatively affect the fitness of the stocked fish in the wild [5–7], the acutely low survival of stocked fish may be largely explained by physiological, behavioral and morphological conditioning to simple hatchery tanks that lack many elements of the natural environment [8–10]. Because many antipredatory and food acquisition skills of fish are learned through ontogeny [11–13], exposing the hatchery fish to natural challenges is expected to improve the stocking success by better preparing the fish for natural environments.

Environmental enrichment, that is, addition of physical structures and complexity in the rearing tanks, has been introduced as a potential measure to improve the welfare of fish and their subsequent post-stocking survival in natural environments [14, 15]. Enrichment can improve cognitive functions and affect explorative behavior with potential consequences on post-release space use [16], but there is no general theory on how structural enrichment would affect traits such as migration or vulnerability to fishing, yet these traits are central in fisheries context. In salmonids that spend their early life in structurally complex riverine environments, structural diversification of tanks has been found to improve, for example, foraging behavior [17], and brain growth, neural plasticity and cognitive ability [18–20]. Yet, the strength and direction of the effects may depend on the species, age of the fish, the type, quantity and duration of enrichment, and other technical factors such as fish density in rearing tanks [21–23]. A few studies have experimentally addressed the effects of enriched rearing on the actual stocking success or vulnerability of fish to fishing [24–26].

Landlocked Atlantic salmon (*Salmo salar* m. *sebago* Girard) in the Finnish Vuoksi watercourse represents a critically endangered salmonid population that has relied virtually entirely on a hatchery breeding program and intensive releases of 2- and 3-year-old fish since the 1970’s due to damming of the original migration routes and redirection of water from an original major spawning river for hydropower production [27, 28]. Regardless of recent habitat restoration actions and complete fishing ban of wild landlocked Atlantic salmon in the Lake Saimaa water system [29, 30], artificial propagation of the population will still be needed for an extended period of time to avoid the imminent risk of the population’s extirpation. Salmon from this strain are also commercially produced and stocked at 2 or 3 years of age to a number of other inland waterways in Finland, mainly to promote recreational fisheries without intentions to establish self-sustaining populations. Most salmon of this population reach their readiness for lake migration (smoltify) by the age 2 and at size of 17–19 cm [27, 31]. Precocious male maturity may occur in 1-year-old and older parr, and maturation of migrating fish occurs at the earliest after two years in lake (at size 65–90 cm and at average age of 5.5 years) [27, 30]. Stocking results have generally been poor with a few exceptions [32], highlighting the need for studies on advanced hatchery management practices.

The first objective of this study was to investigate whether and how the fish reared in enriched tanks, involving submerged shelter structures and periodically changing water inflow patterns, would differ from their counterparts reared in standard tanks with respect to early survival (until 3 years of age), growth and body condition, incidence of fin damages, tendency for downstream smolt migration, and vulnerability to angling. In applied terms, the primary aim was to study whether enriched rearing would bring benefits that would entitle the increased use of enriched rearing practices in the production of landlocked Atlantic salmon smolts in hatcheries. Second, based on tag returns by recreational fishers after experimental stockings into a large lake, we evaluated whether the rearing method would affect the capture rate of the fish in relation to their release age (2 or 3 years) and size or the measured smolt migration parameters. Due to underdeveloped general theory and contradictory findings in literature regarding the manifold effects of enriched rearing on fish, and on salmonids in particular, unidirectional predictions for the expected effects could not be formed for some of the traits. However, we expected that the applied diversification of the environment would improve the survival of fish during rearing [33, 34], reduce growth heterogeneity and fin erosions through decreased level of stress and aggressive encounters [35, 36], decrease vulnerability to experimental angling but increase the vulnerability to lake fisheries through increased foraging and (or) exploration rate [14, 16, 17, 25], and consequently also increase tag return rates, along with possibly decreased natural mortality post release [20]. Lastly, we investigated whether high smolt migration activity would increase individual’s probability to become captured in lake fisheries, as general exploration might increase vulnerability to fishing [25].

## Materials and methods

### Experimental fish and their treatment

**Fig 1:**
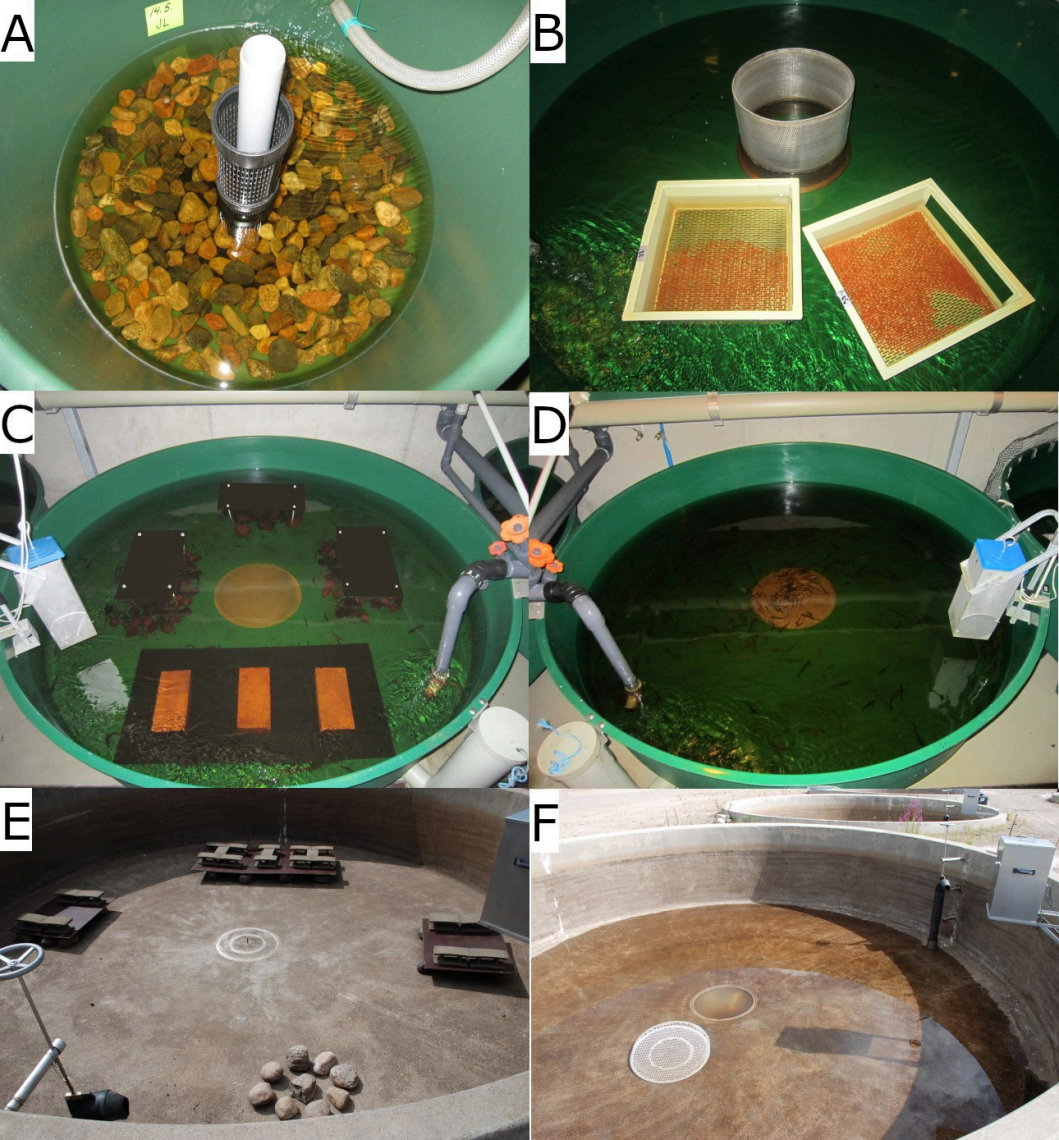
Enriched (left) and standard (right) rearing tanks during egg incubation (A–B), first year of living (juvenile stage; C–D), and on-growing (until smolt stage; E–F). Enriched and standard tanks at different stages of rearing.

This study was conducted during 2009–2015 using offspring from parent fish captured under the lowermost hydropower dam in the original spawning river, River Pielisjoki, Eastern Finland (WGS84 coordinates: 62°42’N, 29°52’E). As a part of governmental responsibility of the Natural Resources Institute Finland (Luke) to establish captive broodstocks using gametes collected annually from wild-caught landlocked salmon spawners, factorial mating of 64 females and 25 males took place in October 2008 at the Saimaa Fisheries Research and Aquaculture Station (62°5’N, 28°54’E) maintained by Luke, Enonkoski. The fertilized eggs were incubated in natural water temperatures at Saimaa station until eyed stage, when approximately 40,000 eggs were transported to the Kainuu Fisheries Research Station (Luke; www.kfrs.fi; 64°24’N, 27°30’E), Paltamo, and equally divided into enriched and standard (plain) rearing environments (23 April 2009). In enriched rearing, the eggs (20,000) were incubated in 0.4 m^2^ tanks (*n* = 8 tanks; 6,250 eggs m^-2^) with 5 liters of gravel (20–60 mm) on the bottom of each tank (Fig 1A), whereas in standard rearing the eggs (20,000) were kept on eight incubation trays set into a 3.2 m^2^ tank (6,250 eggs m^-2^) because it was not feasible to use the same sized tanks for both treatments (Fig 1B). The number of fish used in different phases of the study is summarized in Table 1.

**Table 1:** Rearing procedure of landlocked salmon.

Period	State	Location	Rearing	No. of tanks	Tank size (m^2^)	No. of fish start^†^	No. of fish end^†^	fish m^-2^ end	kg m^-3^ end
**23 Apr–25 May 2009**	From eyed egg to yolk sac	Indoors	Standard	1 (8^a^)	3.2 (0.16^b^)	20,000	18,976	5,930	NA
			Enriched	8	0.4	20,000	15,360	4,800	
**26 May 2009–10 May 2010**	1^st^ year from start feeding	Indoors	Standard	4	3.2	15,360	9,200	719	24.76
			Enriched	4	3.2	15,360	11,964	935	34.03/247.52
**11 May 2010–2 Jan 2011**	2^nd^ year rearing	Outdoors	Standard	4	50	9,200	8,790	44	2.71
			Enriched	4	50	9,200	8,511	43	2.59/7.78
**3 Jan–18 May 2011**	Fish for release at age 2	Outdoors	Standard	5	50	5,000	4,989	20	1.23
			Enriched	5	50	5,000	4,993	20	1.21/3.64
**3 Jan 2011–8 Mar 2012**	3rd year rearing	Outdoors	Standard	1	50	3,641	3,778	73	10.85
			Enriched	1	50	3,421	3,558	67	12.27/36.81
**9 Mar–28 Mar 2012**	Tagging	Indoors	Standard	2	15	1,000	1,000	33	7.3
			Enriched	2	15	1,000	1,000	33	9.2
**28 Mar–31 May 2012**	Fish for release at age 3	Outdoors	Standard	1	50	500	500	10	1.43
	(only T-tag)		Enriched	1	50	500	500	10	1.73/5.18
**28 Mar–27 Apr 2012**	Fish for migration and stocking	Outdoors	Standard	1	50	500	500	10	1.43
	experiments 2012 (T- & PIT-tag)		Enriched	1	50	500	500	10	1.73/5.18
**27 Apr–28 May 2012**	Fish in migration test (T- & PIT-tag)	Outdoors	Standard	4	39	500	491	3	1.72
			Enriched	4	39	500	495	3	2.18
**22 Aug–2 Sept 2012**	Experimental angling, mixed ponds of PIT-tagged fish	Outdoors	Standard Enriched	2	400	199	199	0.25	0.15

Description of the rearing procedure.

^a^Decrease from start to end of rearing period includes mortality and fish taken for other use

^b^Eggs from standard rearing were set on 8 × 0.16 m^2^ grids (20 × 3.5 mm mesh size) on the bottom of one 3.2 m^2^ tank.

^c^For enriched rearing, two rearing density means are given for alternative water volumes due to applied changes in water level (see Table 2).

Following complete hatching (26 May 2009), the rearing densities were equalized, and the fish (alevins) were divided into 3.2 m^2^ indoor tanks made of dark green fiberglass (Fig 1C and 1D). In the beginning, there were 15,360 0-year-old fish in both rearing treatments (four tanks per rearing method, 3,840 fish per tank, 1,200 fish m^-2^). Following Hyvärinen and Rodewald [24], standard tanks were kept plain without any structures or substrate, whereas enriched tanks were furnished with stones and overhead shelters of different size (Fig 1C and 1E). Stones change the bottom structure and serve as natural shelters for juvenile salmonids. Overhead covers in turn provide fish with shades to hide and thereby expectedly decrease stress. Further, the water level, and direction and velocity of the currents were altered irregularly (time interval 3–5 days) affecting the habitat features and food dispersal. For example, the low water level caused the feed to settle to the bottom of the tanks quickly, and the fish had to learn to look for food also on the bottom. Under standard conditions, fish may learn only one type of feeding behavior close to the surface, because the food always comes in the same way. The enrichment structures were adjusted during rearing to match the growth of fish (Table 2).

**Table 2:** Water depth, velocity and direction changes, and shelter structures used for the enriched rearing. There was no variation in these factors for the standard rearing.

Time	Rearing	Alternative	Water depth (cm)	Water velocity (l s^-1^)	Water direction	Shelter^a^
**23 Apr–25 May 2009**	Standard	1	11	0.3	Clockwise	none
**no changes**	Enriched	1	11	0.2	Clockwise	gravel^b^
**26 May–13 Jul 2009**	Standard	1	11	0.4	Clockwise	none
**3–5 days between changes**	Enriched	1	11	0.4	Clockwise	gravel + cover
		2	11	0.4	Counterclockwise	gravel + cover
**14 Jul–20 Oct 2009**	Standard	1	20	0.6	Clockwise	none
**4–24 days between changes**	Enriched	1	11	0.5	Clockwise	gravel + cover
		2	11	0.5	Counterclockwise	gravel + cover
		3	20	1.4	Clockwise	gravel + cover
		4	20	1.4	Counterclockwise	gravel + cover
**21 Oct 2009–10 May 2010**	Standard	1	25	0.9	Clockwise	none
**4–24 days between changes**	Enriched	1	11	0.7	Clockwise	gravel + cover
		2	11	0.7	Counterclockwise	gravel + cover
		3	11	0.7	Directed to middle of tank	gravel + cover
		4	25	1.6	Clockwise	gravel + cover
		5	25	1.6	Counterclockwise	gravel + cover
		6	25	1.6	Directed to middle of tank	gravel + cover
**11 May 2010–31 May 2012**	Standard	1	115	7.3	Clockwise	none
**7–44 days between changes**	Enriched	1	40	5.0	Clockwise	stones^c^ + cover
		2	40	5.0	Counterclockwise	stones + cover
		3	40	5.0	Directed to middle of tank	stones + cover
		4	120	10.4	Clockwise	stones + cover
		5	120	10.4	Counterclockwise	stones + cover
		6	120	10.4	Directed to middle of tank	stones + cover

Enrichment attributes used at different stages of enriched rearing.

^a^Size and material of cover: 26 May–22 Aug 2009: three sheets of 2 mm × 500 mm × 250 mm polystyrene with metal legs. 23 Aug 2009–10 May 2010: one sheet 2 mm × 500 mm × 1000 mm PVC with bricks (60 mm × 120 mm × 260 mm) beneath and on top, three sheets of 2 mm × 500 mm × 250 mm polystyrene with metal legs. 11 May 2010–31 May 2012 two sheets of 1500 mm × 1500 mm plywood, one sheet of 1500 mm × 3000 mm plywood with stones (200–400 mm) beneath and bricks (390 mm × 200 mm × 190 mm) on top.

^b^Gravel size approx. 30–80mm.

^c^Stone size approx. 200–400mm.

Fish densities were equalized for enriched and standard rearing groups again on 11 May 2010 when the 1-year-old fish were moved outdoors into 50 m^2^ concrete tanks (Fig 1E and 1F). In the start of the outdoor rearing period, there were 9,200 fish in standard rearing (4 tanks, 2,300 fish per tank, 46 fish per m^2^) and 9,200 fish in enriched rearing (4 tanks, 2,300 fish per tank, 46 fish per m^2^). The variation of the inflow continued in enriched tanks also outdoors, and the size of the shelters and stones was adjusted to fit the size of the fish.

After the second summer on 16 November 2010 the age 1+ fish were moved indoors and tagged individually (22 November–9 December 2010) for subsequent stocking experiments. At tagging, approximately 1,000 fish per rearing method were haphazardly sampled from each of the four replicate tanks, sedated with clove oil (70–80 mg l^-1^) and equipped with a T-bar anchor tag (Hallprint Pty Ltd.). Simultaneously with tagging, total length (TL) was measured in millimeters and body weight (BW) to the nearest 0.1 g on all fish. The adjusted condition factor (CF) was calculated using the equation: CF = 100 × BW (g) × (TL (cm))^*-b*^, where *b* (2.93) is the slope of a nonlinear regression of BW on TL from the pooled data of individual measurements [37]. Further, the condition of dorsal fin, both pectoral fins and both pelvic fins was visually assessed from each tagged fish and classified on a scale of 0 to 4: 0 = pristine, 1 = erosion <25%, 2 = erosion approx. 50%, 3 = erosion approx. 75%, 4 = full erosion. Fin damages, together with fish growth (and size variation), were used as an indication of internal tank aggression [38, 39]. Precociously matured males were identified based on their external characteristics and running milt.

Nearly one month after the tagging had been completed (3 January 2011), the 2-year-old fish were moved back to the outdoor tanks. There were 5 × 50 m^2^ concrete tanks for standard rearing and 5 × 50 m^2^ concrete tanks for enriched rearing with 1000 tagged fish (T-tag) in each tank (20 fish per m^2^). In addition, there was one 50 m^2^ standard tank (3,778 fish, 76 fish per m^2^) and one 50 m^2^ enriched tank (3,641 fish, 73 fish per m^2^) with untagged fish to be used in the migration and stocking experiments in 2012 (see below). The untagged fish were kept in their rearing tanks until 9^th^ March 2012 when they were moved indoors for another set of tagging.

Tagging of 3-year-old (hereafter 3-yo) fish was performed 12–28 March 2012: 500 standard and 500 enriched-reared fish were tagged using T-bar anchor tag, and other 500 standard and 500 enriched-reared fish were tagged with both T-bar anchor tag and a half duplex PIT-tag (ventrally implanted into the body cavity; 23 × 4 mm; Texas Instruments Inc., http://www.ti.com). During tagging, individual data on TL, BW, fin erosions and sexual maturation were collected as previously. Because no replicate tanks were used for the rearing methods at that time, the measurements were not analyzed further (for the basic statistics, see S1 Table). After tagging, the fish were moved back to four 50 m^2^ outdoor concrete tanks (28 March 2012; each tagging group in their own tank) to be reared until the migration, angling and stocking experiments.

Variation in water temperature through the rearing period (23 Apr 2009–30 May 2012) is shown in S1 Fig. During the first year of rearing, indoor lighting was adjusted to follow natural photoperiod. Starting from 30 May 2009, all the fish were fed commercial dry feed (Nutra ST and Vital Plus, Raisioaqua Ltd) of growth-adjusted size using automatic feeders. Dead fish were recorded and removed from the tanks on a daily basis. In accordance with routine hatchery practices, formalin was used as a bath treatment to control external parasites of the fish, whereas bacterial infections were treated with antibiotics (Orimycin vet). Diseases were diagnosed by the Finnish Food Authority and multicellular parasites through microscopic inspections at the station.

The experiment was conducted in accordance with national regulation for treatment and welfare of experimental animals under licenses n:o ESLH-2008-04178/Ym-23 and ESAVI/2458/04.10.03/2011 granted by the Animal Experiment Board in Finland (ELLA). For captures of spawners, permissions were provided by the regional fisheries authority, North Carelia T&E Centre (license number 1631/5716/07), and the landowner, Pohjois-Karjalan Sähkö Oy (field site access).

### Experimental determination of individual smolt migration patterns

To study whether the smolt migration tendency (downstream) differed between enriched and standard fish, the 3-year-old individuals tagged with both T-tag and PIT-tag were divided into eight circular channels with concrete walls and gravel bottom on 27 April 2012 (125 fish per channel, four channels per rearing method; outer circumference 30.87 m, inner circumference 21.44 m, channel width 1.50 m, water depth approx. 33 cm, water flow 0.09 m s^-1^) [40]. Migration behavior was monitored using PIT-telemetry and four equally distributed looped submerged antennas from 27 April 2012 at 13:00 to 28 May 2012 at 13:00. PIT data were first transformed to quarterly movements upstream, downstream or to unidentified direction per hour based on the order of PIT detections by the four antennae (http://www.pitdata.net) [40, 41]. Between-antenna movements were transformed to distances based on the information that each full round corresponds to 26.15 m as measured in the middle of the channel. The hourly data were further analyzed for each PIT id by using custom codes in AV Bio-Statistics 5.2 (written by A. Vainikka). We calculated for each fish 1) the total distance moved downstream in meters, 2) percentage of movement oriented downstream, 3) hour when the total movement started to increase most rapidly during the 72 hour moving window as estimated by linear regression slope, 4) hour of the beginning of the time window when the moving average (72 hours) was the highest, 5) maximum total activity average during the 72 hour window, and 6) average total activity during the whole test period.

### Experimental angling

On 5 July 2012, 199 3-year-old salmon, measured for body size at tagging in March 2012 (98 enriched-reared fish, mean TL 277 ± 19 (SD) mm, mean BW 208.3 ± 46.2 g, and 101 standard-reared, 267 ± 16 mm, 171.3 ± 59.2 g) and tagged with both T-bar anchor tags and PIT-tags, were transported to a temporary 50 m^2^ (*n* = 100–102 fish in each) concrete tank from which they were further transferred to two 400 m^2^ earth-bottomed ponds (maximum depth approx. 1.5 m) for experimental angling that took place between 22 August 2012 and 2 September 2012 with two days break 27–28 August 2012. The fish were manually fed (60 g of dry feed per pond) each evening (19:00) prior to next day’s fishing that was conducted with a randomized set of fly patterns (1 dry flies, 2 muddler and 3 tinsel type streamers, 4 woolly buggers, 5 nymphs and 6 larvae/pupa, two fly patterns per category) using AFTMA #5 Vision GT4 rod, floating line and sink-tip fly-fishing technique. The fishing took place in periods of 1 hour during which the ponds were fished alternatively 15 min from opposite sides to let the fish in the other pond recover from the disturbance prior to the next try. The used fly pattern within the fly category was changed after 30 min (while the fly type was always the same for the whole hour). Due to the low realized capture rates, all possible fly patterns were used on the last day of experiments in an order chosen by the test angler (September 2 2012). All fly patterns were tied to #6 - #16 size barbless hooks. Each day, the fishing took place at 10:00, 14:00 and 18:00, and was performed by a single experienced fly fisher. A fish landed using a landing net was identified by its PIT-tag, recorded for the time of the capture and returned to the pond immediately. No sedation was needed. One fish died to deep hooking. Three fish (2 enriched, 1 standard) hooked from other position than mouth were included in the captures. Two fish were captured twice, but only their first capture was used in the analyses. For each fish, the total time until captured was calculated in days from the start of the fishing and used in the analysis (maximum duration was assigned for the non-captured fish). To account for the effect of fish size and condition on the angling results, all fish were measured for their TL and BW after the experiment (5 Sept 2012). Condition factor (CF) was calculated for the initial and final measurements using common empirical allometric exponent as CF = 10^6^ BW/(TL^3.452^). Change in condition factor between March and September was calculated as ΔCF = CF_Sept_−CF_March_.

### Fish releases and tag returns from lake fisheries

To investigate whether environmental enrichment would increase capture rates in lake fisheries, as indicative of good stocking success, over standard-reared fish, individually tagged fish were stocked at two (hereafter 2-yo fish) and 3 years of age (3-yo) into a large clear-water Lake Höytiäinen, Eastern Finland (62°49’N, 29°39’E; area 283 km^2^, mean depth = 11.8 m, maximum depth = 56 m). Lake Höytiäinen is intensively fished both commercially and recreationally, and supports small pelagic fish, European smelt (*Osmerus eperlanus*) and vendace (*Coregonus albula*) for predatory salmonids to feed on. The predatory fish community consists of an abundant but small-bodied pikeperch population, and small burbot and a scarce pike population that were assumed to pose a marginal predation threat for the stocked salmon [40]. The 2-yo fish were transported on 20 May 2011 from the Kainuu Research Station to two release sites situated on the eastern (Saunasaari) and western side (Kontioniemi) of the lake. The number of 2-yo fish released was lower in enriched rearing group (*n* = 1531 fish) than in standard rearing group (*n* = 1909) due to accidental water leak in one transport tank and consequential mortality (all the survivors released, 74% of the enriched-reared group). The 3-yo fish were released on 30 May 2012 in Kontioniemi (*n* = 895 enriched and 894 standard fish), including all PIT-tagged fish that were used in the migration test but not in angling experiment.

Information on voluntary tag returns was collected to the national tagging database maintained by Luke. Returning of the fish tags is encouraged by a small monetary reward, but the returns do not cover 100% of the captured fish. Along with tag returns the fishermen are asked to report some information about their catch such as the date and site, fishing method used, and the length and weight of the fish. The fishing methods in this study were grouped into three categories: 1) hook and line gears involving different forms of rod fishing, i.e. trolling and spin fishing, 2) stationary gears involving gillnets and fyke net fishing, and 3) unknown gear (no information given). There were no tag returns for either of the age groups after the year 2015 (situation checked in September 2019). Experimental releases of fish were performed under the fishing law (286/82) and the required permission from the local fisheries region to stock strain of fish that already was present in the receiving waterbody.

### Statistical analyses

All analyses were performed using SAS 9.4 (SAS Institute, Cary, NC, USA) and IBM SPSS Statistics 27.0.1.0 (IBM, Armonk, NY, USA). First, differences of means in BW and CF were tested between standard and enriched groups using data on 2-summer-old fish only. A linear mixed model (MIXED procedure in SAS) with Restricted Maximum Likelihood estimation method was:

$$y_{ijk}=\mu +Rearing_{j}+Tank_{k\left(j\right)}+e_{ijk},$$ (1)

where *y*_*ijk*_ is a body trait of an individual *i*, *μ* is the population mean, Rearing_*j*_ is the fixed effect of rearing method (*j* = 1–2), Tank_*k(j)*_ is the random effect of rearing tank, nested within rearing method (*k* = 1–4), and *e*_*ijk*_ is the random error term. Separate error variances, one for each rearing method, was fitted in the model of BW, whereas a single common variance across the treatment groups was fitted for CF. The goodness of fit of the alternative models including a common or separate error variances was compared using the likelihood ratio test [42]. Standard errors and degrees of freedom for the tests of the fixed effects were calculated using the method by Kenward and Roger [43].

The probabilities of having fin erosions were compared between the rearing methods using a generalized linear mixed model for multinomial data (GLIMMIX procedure in SAS; ordinal scale 0–4 for each fin). The model was separately tested for dorsal fin, right and left pectoral fins, and right and left pelvic fins using cumulative logit link function and a residual pseudo-likelihood (RSPL) estimation method. The terms in this model were the same as given above (1).

Principal Component Analysis (PCA) with varimax rotation was used to reduce different migration activity measures (six variables, see above) into uncorrelated principal components (PC) having eigenvalue larger than one. To fulfill the assumption of normal distribution, ln(*X* + 1)-transformation was used for both time variables (3 and 4, see above). SAS automatically standardizes all input variables. Then, to analyze whether there were differences between the standard and enriched rearing groups in their obtained principal component scores, the following linear mixed model was fitted to the data on individual PC scores:

$$y_{ijlm}=\mu +Rearing_{j}+Maturity_{l}+Channel_{m\left(j\right)}+e_{ijlm},$$ (2)

where the fixed effect of sexual maturation (*l* = 0 or 1), and random variance due to test channel (*m* = 1–4), nested within rearing method, were taken into account. In addition, to correct for the potential effect of body size on migration tendency, fish TL was included in the model as a fixed covariate (regression term). Prior to inclusion of the covariate in the models, covariate × factor interactions were tested (non-significant in each case). Asymptotic Kolmogorov-Smirnov (K-S) two-sample test was used to evaluate whether the distributions of PC scores differed between the rearing groups. Here, Monte Carlo estimation with 10,000 samples was used to compute exact *p*-values.

Vulnerability to experimental angling was studied using Cox regression with rearing treatment as a factor and fish TL and CF or ΔCF as continuous explanatory variables and backward log-likelihood ratio-based model selection. Pond information was not available individually, but due to the even captures (15 in pond 1 and 18 in pond 2) no pond effects were expected. Cox regression was also used to analyze the risk to become captured separately in the lake fisheries. The model was fitted separately for the stocking age groups using rearing method, stocking site (for 2-yo fish) and their interaction as factors and fish tagging length as a continuous variable. Further, the time until capture and fish length at capture were examined using linear mixed-effects models among the captured fish. The response variable ‘days until caught’ was normalized before analysis using Box-Cox transformations (λ = 0.5 for 2-yo fish and λ = -0.25 for 3-yo fish). The used linear mixed model, fitted separately for the age groups, was:

$$y_{ijop}=\mu +Rearing_{j}+Gear_{o}+Rearing_{j}\times Gear_{o}+Site_{p}+e_{ijop},$$ (3)

where Gear_*o*_ is the fixed effect of fishing method category (*o* = 1–3; hook and line, stationary or unknown gear type), Rearing_*j*_ × Gear_*o*_ is the interaction between rearing and fishing methods, and Site_*p*_ is the random effect of release site (*p* = 1–2; used only for 2-yo fish). Further, to correct for the potential effects of initial fish size on both response variables, fish tagging length was included in the model as a fixed covariate (and used when significant). In addition, for capture time, the effect of fish CF at tagging was tested as another covariate. The least square means obtained from the models were further compared between the rearing methods using pairwise *t* tests with Tukey-Kramer adjusted *p*-values separately for each fishing method category.

Lastly, we tested whether an individual’s migration activity in experimental channels predicted its probability to be captured in lake. This test leans on an assumption that some pattern of smolt migration such as high total activity might also reflect activity in lake, thereby being (positively) associated with capture probability. Here, a generalized linear mixed model for individual binary data (captured or not) was used. This model included the rearing method, obtained PC scores (covariate) and their reciprocal interaction terms as fixed predictors. The association between PC scores and capture days was analyzed for each rearing method separately using Pearson’s correlation analysis. The threshold for statistical significance was considered as *p* = 0.05 in all analyses.

## Results

### Survival during hatchery rearing

**Fig 2:**
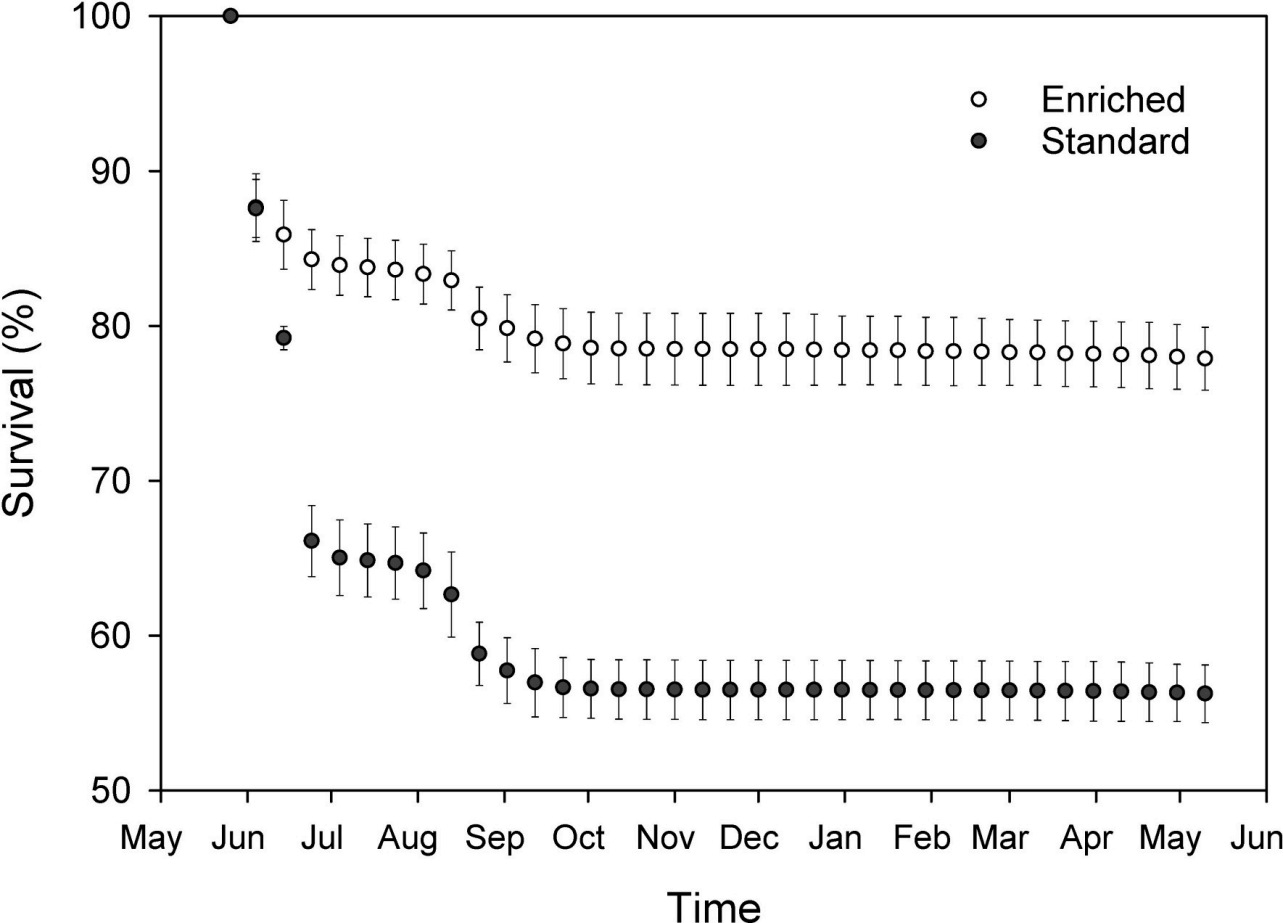
Survival rates of landlocked salmon during the first year (mean ± SD%, *n* = 4 tanks per rearing method; period: 26 May 2009–11 May 2010) in enriched and standard rearing tanks. For clarity, values are only given for every tenth day. Survival rates of enriched- and standard-reared salmon during the first year.

During the first year, survival was substantially higher in enriched (mean = 77.9%, range = 76.0–80.8%, *n* = 4 tanks) than in standard rearing (mean = 56.2%, range = 54.9–59.0%, *n* = 4 tanks) (Fig 2). Mortality was primarily associated with parasitic (*Ichthyobodo necator*) and bacterial (*Flavobacterium* sp.) infections during one epidemic in June and another in August–September (Fig 2). Since the group size equalization in May 2010, the survival rate until 3 January 2011 was high and, on average, similar in enriched (mean = 93.6%, range 91.0–95.8%, *n* = 4 tanks) and in standard rearing (mean = 96.3%, range 95.1–97.0%, *n* = 4 tanks). During this period, white spot disease caused by *Ichthyophthirius multifiliis* parasite was the main cause of mortality. Since tagging and the second equalization of the group sizes to 1000 fish per tank (3 January–20 May 2011), survival ranged from 99.3 to 100% in both rearing environments. Survival was 98.5% in the enriched group and 91.7% in the standard group among the fish reared until the age 3 (3 January 2011–12 March 2012).

### Growth, condition and maturation

The mean BW of fish did not differ significantly between the rearing methods after two summers of growing (linear mixed-effects model: *F*_1,5.79_ = 3.80, *p* = 0.101; least square mean ± SE: 70.8±0.8 g in standard rearing, 73.0±0.8 g in enriched rearing). Phenotypic variation in BW was marginally higher in enriched (CV: 34.7%) than in standard group (CV: 28.4%). This observation was supported statistically by the improved model fits using separate error variances (likelihood ratio test; *G*^2^ = 209.9, *p* < 0.001). The mean CF was slightly (yet significantly) higher in enriched (1.24±0.01) than in standard group (1.18±0.01; *F*_1,6_ = 32.52, *p* = 0.001).

Dorsal fin showed the most fin erosion: The degree of erosions was higher in the enriched group (*F*_1,6_ = 4.71, *p* = 0.073; Table 3). The odds of the standard group displaying lower dorsal fin erosion category was approximately 1.8 times the odds of the enriched group. Dorsal and pelvic fins were mainly intact in both rearing methods (Table 3), though the difference of erosion levels in the left pelvic fin approached statistical significance between the rearing groups (*F*_1,6_ = 4.68, *p* = 0.074), i.e., higher degree of damages found in the enriched group. The proportion of mature 2-summer-old males was 0.2% in both rearing groups.

**Table 3:** Number of 2-summer-old salmon (% in brackets) in standard and enriched rearing groups assigned to each fin erosion category.

		Fin erosion score^a^				
Fin		0	1	2	3	4
**Dorsal**	Standard	896 (22.4)	978 (24.5)	1,021 (25.5)	912 (22.8)	190 (4.8)
	Enriched	583 (14.6)	811 (20.3)	915 (22.9)	1,398 (35.0)	291 (7.3)
**Right**	Standard	3,861 (96.6)	101 (2.5)	29 (0.7)	5 (0.1)	1 (0.0)
**pectoral**	Enriched	3,791 (94.8)	189 (4.7)	14 (0.4)	3 (0.1)	1 (0.0)
**Left**	Standard	3,903 (97.7)	76 (1.9)	15 (0.4)	2 (0.1)	0 (0.0)
**pectoral**	Enriched	3,782 (94.6)	196 (4.9)	13 (0.3)	5 (0.1)	3 (0.1)
**Right**	Standard	3,909 (97.8)	79 (2.0)	8 (0.2)	1 (0.0)	0 (0.0)
**pelvic**	Enriched	3,627 (90.7)	208 (5.2)	118 (3.0)	38 (1.0)	8 (0.2)
**Left**	Standard	3,905 (97.7)	78 (2.0)	11 (0.3)	3 (0.1)	0 (0.0)
**pelvic**	Enriched	3,562 (89.1)	206 (5.2)	104 (2.6)	80 (2.0)	47 (1.2)

Number of enriched- and standard-reared 2-summer-old salmon in different fin erosion categories.

^a^0 = pristine; 1 = erosion <25%; 2 = erosion approx. 50%; 3 = erosion approx. 75%; 4 = full erosion.

No statistical comparison could be made between the rearing groups at 3 years of age due to the lack of replicate tanks. Yet, the fish in enriched environment showed higher mean and variation in TL, BW and CF compared to the standard-reared fish (S1 Table). In addition, the differences in the degree of erosion in dorsal fin aligned with the results at the age 2 by being higher in the enriched- than standard-reared group (S2 Table).

### Migration activity (3-yo)

**Fig 3:**
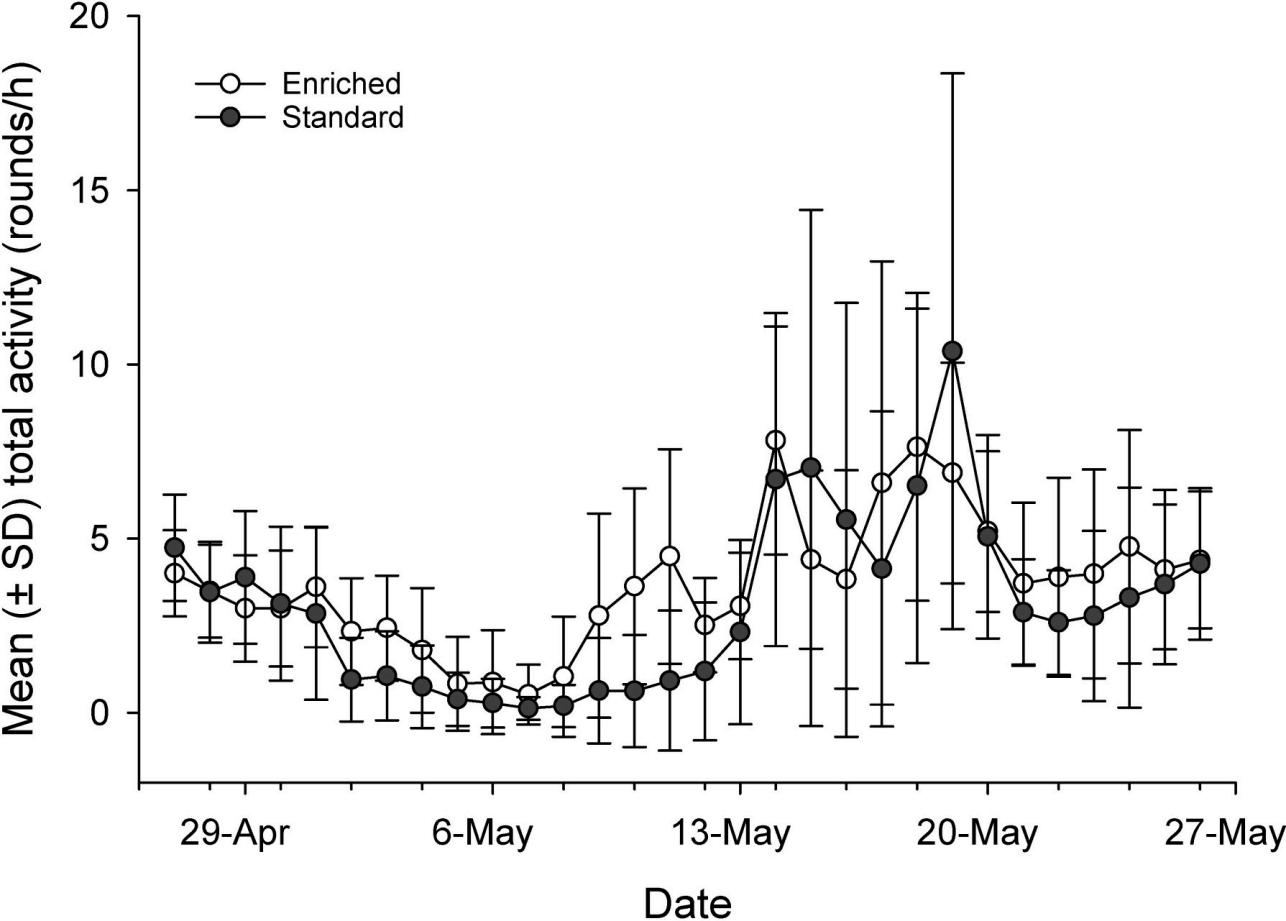
Mean ± SD of average daily activities in enriched- (*n* = 495) and standard-reared fish (*n* = 491) over the whole experiment as starting from 13:00 on 27 April 2019 and ending on 28 May at 13:00. Daily activity means in enriched- and standard-reared salmon during the smolt migration experiment.

Movement data were obtained from 491 standard fish and 495 enriched 3-yo fish. In both rearing groups, mean total activity showed a highly fluctuating pattern through the experiment, with large individual variation (Fig 3). PCA resulted in two PCs with eigenvalue higher than one, accounting for 64% of the total variation in the measured behavioral variables. PC1 (42.9% of total variation) was interpreted to reflect the level of total migration activity, whereas PC2 (21.1%) involved the proportion of movements oriented downstream (positive loading) and inversely the onset of maximal migration speed (Table 4).

**Table 4:** Principal components extracted from PCA and migration variables included in the analysis (varimax-rotated component matrix). Three highest loadings per PC are bolded.

Variable	PC1	PC2
**Distance moved downstream**	**0.377**	-0.071
**Percentage moved downstream**	0.059	**0.560**
**Hour when the total movement started to increase most rapidly during the 72 hour window** ^a^	-0.023	**0.480**
**Hour of the beginning of the time window when the moving average was highest** ^*^	-0.024	**0.403**
**Maximum total activity average during the 72 hour window**	**0.324**	0.231
**Average total activity during the whole test period**	**0.369**	-0.159

Principal component loadings for different migration variables.

^a^Ln(X+1) transformation used.

**Fig 4:**
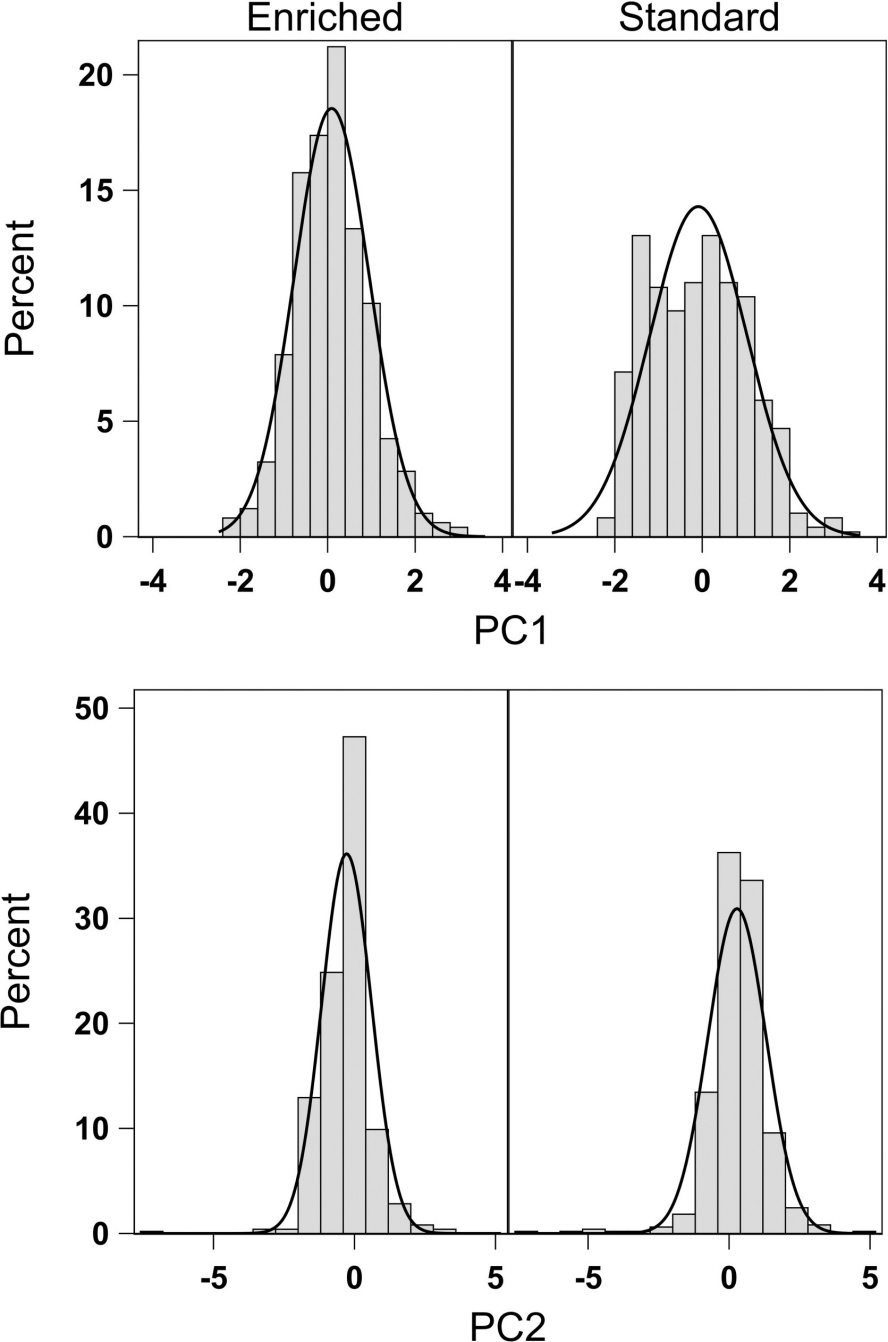
Frequency distributions (with normality curves) of principal component scores depicting migration activity in enriched- (*n* = 495) and standard-reared fish (*n* = 491). Frequency distributions of migratory activity components in enriched- and standard-reared salmon.

The rearing method did not affect either of the PC score means (PC1: *F*_1,6.01_ = 0.04, *p* = 0.856; PC2: *F*_1,6.02_ = 2.40, *p* = 0.172), indicating that both groups showed, on average, similar downstream migration patterns. However, comparison of the PC score distributions revealed statistically significant differences between the rearing groups (K-S test: PC1: D = 0.188, exact *p* < 0.001; PC2: D = 0.350, exact *p* < 0.001). In particular, the kurtosis of PC1 score distribution was clearly higher for enriched-reared fish (0.432) than for standard-reared fish (-0.539), demonstrating a more consistent pattern of total migration activity within enriched rearing group (Fig 4). Variation in the scores of both PCs was significantly affected by sexual maturity in the preceding autumn [PC1: estimate for immature fish 0.277 (95% CI = 0.130–0.425), *F*_1, 974_ = 13.57, *p* < 0.001; PC2: estimate for immature fish -0.213 (-0.376– -0.050), *F*_1, 974_ = 6.55, *p* = 0.011] and covariate fish TL [PC1: estimate 0.011 (0.008–0.013), *F*_1, 974_ = 68.98, *p* < 0.001; PC2: estimate -0.009 (-0.011– -0.006), *F*_1, 974_ = 37.44, *p* < 0.001 in both PCs]. Thus, increasing fish size and immature status increased migration activity and advanced the attainment of maximal migration speed.

### Experimental angling (3-yo)

Out of 199 fish, 33 fish (16 standard- and 17 enriched-reared) were captured at least once. CF had a complex effect on vulnerability to angling, as initial models based on size measures in March or September indicated a marginal positive effect for CF_March_ (estimate 4.42, *p* = 0.097) and a marginal negative effect for CF_Sept_ (estimate -6.66, *p* = 0.068). According to the final model, ΔCF was the only significant term in explaining vulnerability to angling (estimate -8.230±2.684 SE, Wald = 9.403, df = 1, *p* = 0.002). Rearing treatment (estimate -0.447±0.421 for standard rearing, Wald = 1.130, df = 1, *p* = 0.288) and fish TL in September (estimate -0.010±0.006, Wald = 2.746, df = 1, *p* = 0.098) were dropped from the final model as non-significant.

### Tag returns from released fish (2- and 3-yo)

**Fig 5:**
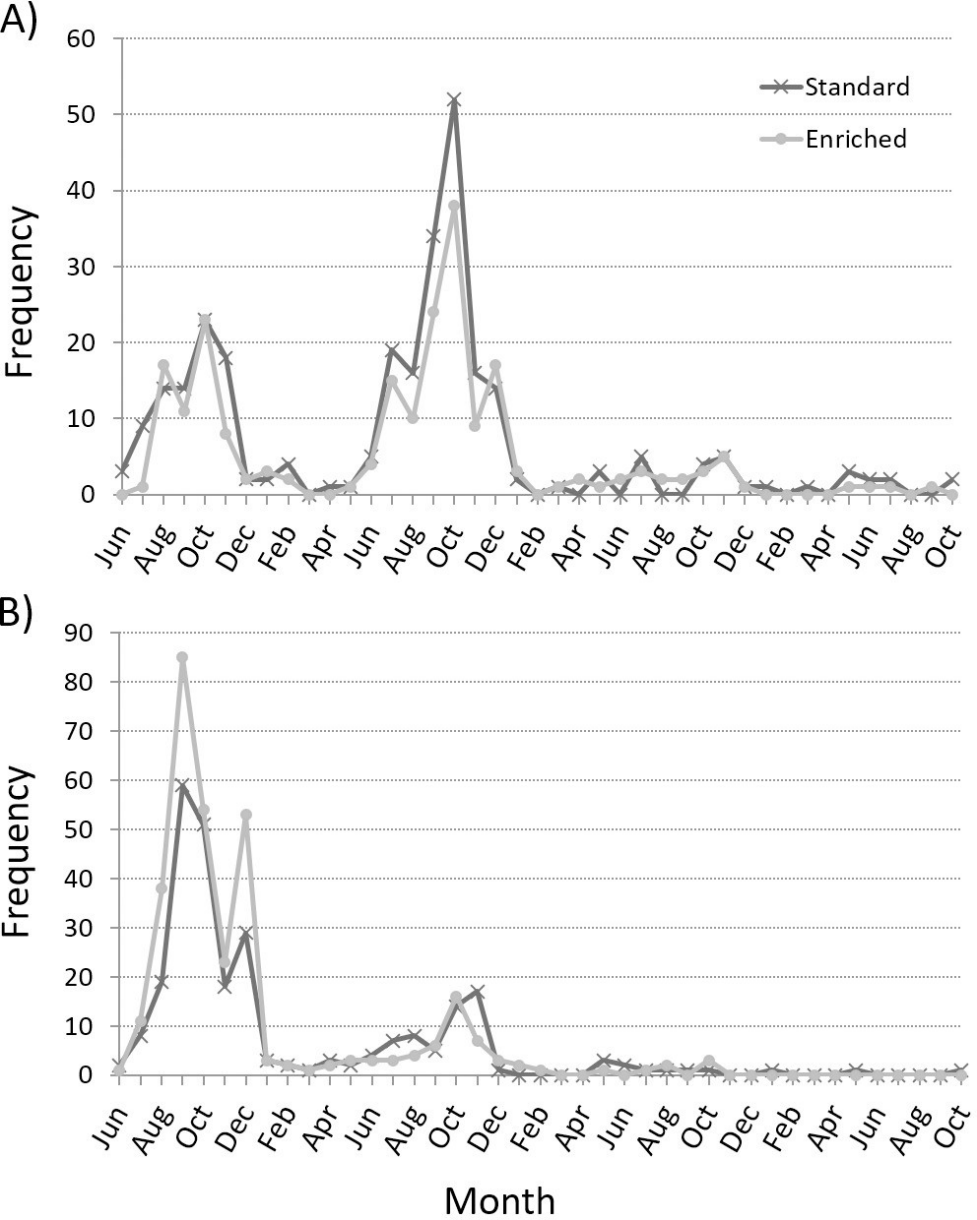
Number of tag recoveries per month for the enriched and standard salmon released into Lake Höytiäinen at age A) 2 (June 2011–October 2014) and B) 3 (June 2012–October 2015). Tag recoveries per month for the enriched and standard salmon from Lake Höytiäinen.

For the fish released at age 2, tag returns were obtained from 490 individuals. 277 tags came from standard-reared fish (14.5% return rate) and 213 from enriched-reared fish (13.9% return rate). Four fish were reported twice (released after the first capture). Hook and line fishing method was reported for 175 fish (36%), stationary gears for 286 fish (58%), and 29 tag returns (6%) had no information about the gear type used. A majority of the tags from 2-yo fish (87.6%) were returned by the end of 2012 (Fig 5A), the average post-release longevity being 430 days (±247 SD; range 36–1243 days). More than half of the fish were caught with gillnets having bar length larger than 40 mm (*n* = 255 2-yo fish). Cox regression model revealed that fish tagging length was positively associated with the risk of capture [estimate 0.025±0.003 SE, Wald χ^2^ = 57.245, *p* < 0.001; hazard ratio for an increase of 10 mm was 1.284 for standard rearing (95% CI 1.203–1.369) and 1.294 for enriched rearing (1.220–1.373)], whereas rearing method or stocking site had no effect.

**Fig 6:**
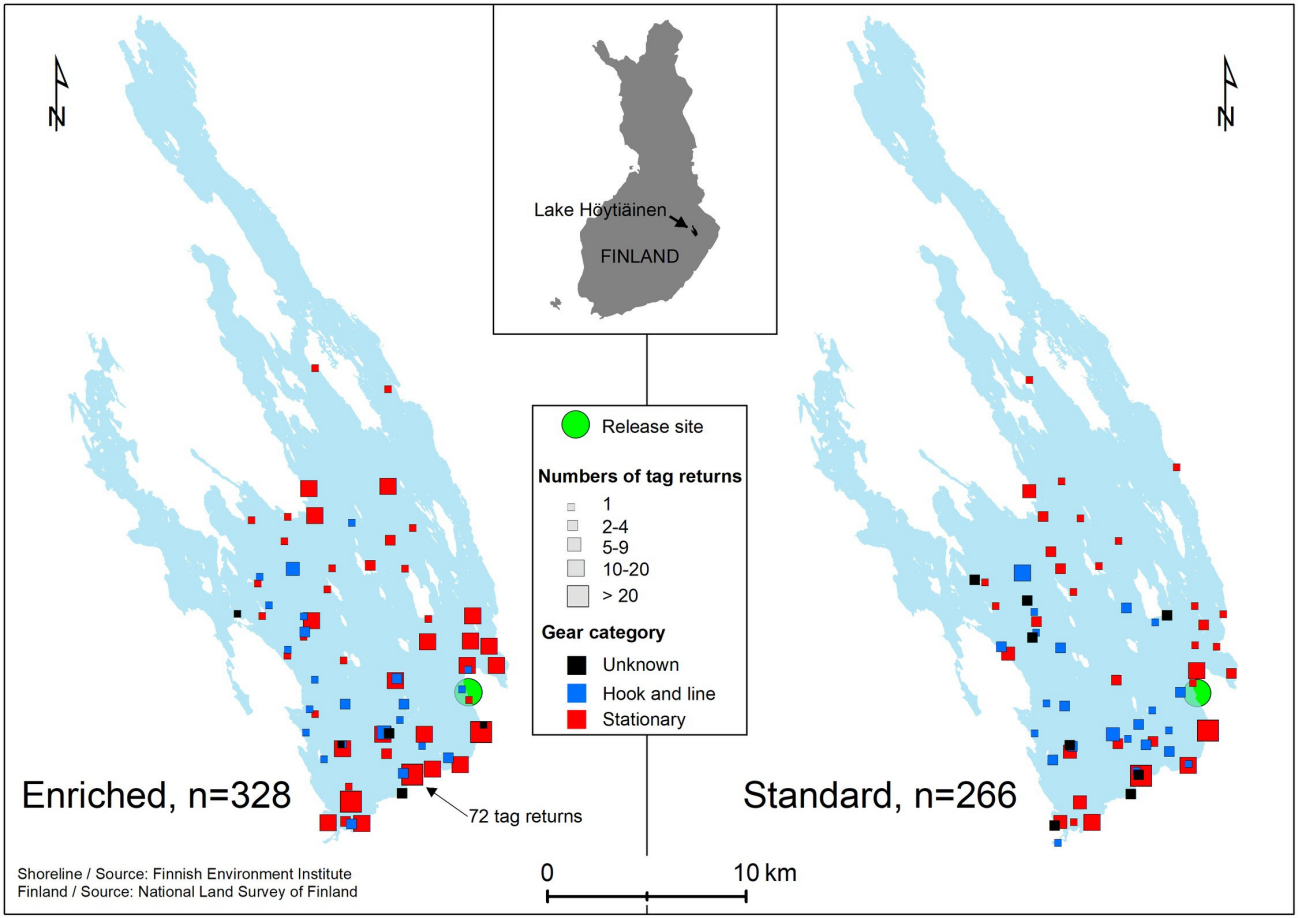
Map graphs of Lake Höytiäinen showing the spatial distribution of tag returns for the released 3-yo salmon in regard to rearing method and gear category. The largest number of returns for one location is indicated on the left-sided graph. The graphs were reprinted using two open information sources from the Finnish Environment Institute (lake shoreline) and National Land Survey of Finland (map of Finland) [44, 45]. Spatial distributions of captures for enriched- and standard-reared 3-yo salmon in Lake Höytiäinen.

For the fish released at age 3, tag returns were obtained from 594 individuals. 266 tags came from standard-reared fish (29.8%) and 328 from (36.6%) enriched-reared fish. Most fish were captured in the southern part of the lake where water depth and clarity are greater (Fig 6). Hook and line fishing method was reported for 100 fish (16.8%), stationary gears for 466 fish (78.5%), and 28 tag returns (4.7%) had missing gear information. Notably more of the 3-yo fish from the enriched group were captured with stationary gears (*n* = 271, 27.3%) than fish from the standard group (*n* = 195, 19.6%). For hook and line gears, the difference to the other direction was less clear (*n* = 45 enriched and 55 standard fish). Most of the 3-yo fish were caught during the stocking year (76.1% in 2012; Fig 5B) with gillnets having bar length larger than 40 mm (73.9%). The average time from release to capture was 221 days (±183 SD; range 14–1249 days). Like in 2-yo fish, tagging length was positively associated with the risk of capture [estimate 0.014±0.004 SE; Wald χ^2^ = 14.905, *p* < 0.001; hazard ratio for an increase of 10 mm was 1.153 for standard rearing (95% CI 1.073–1.240) and 1.061 for enriched rearing (1.013–1.112)]. In addition, enriched rearing significantly increased the risk of 3-yo fish to become captured, in relation to standard rearing [estimate 2.441±1.198 SE, Wald χ^2^ = 4.148, *p* = 0.042; hazard ratio 1.201 (95% CI 1.017–1.417)].

**Fig 7:**
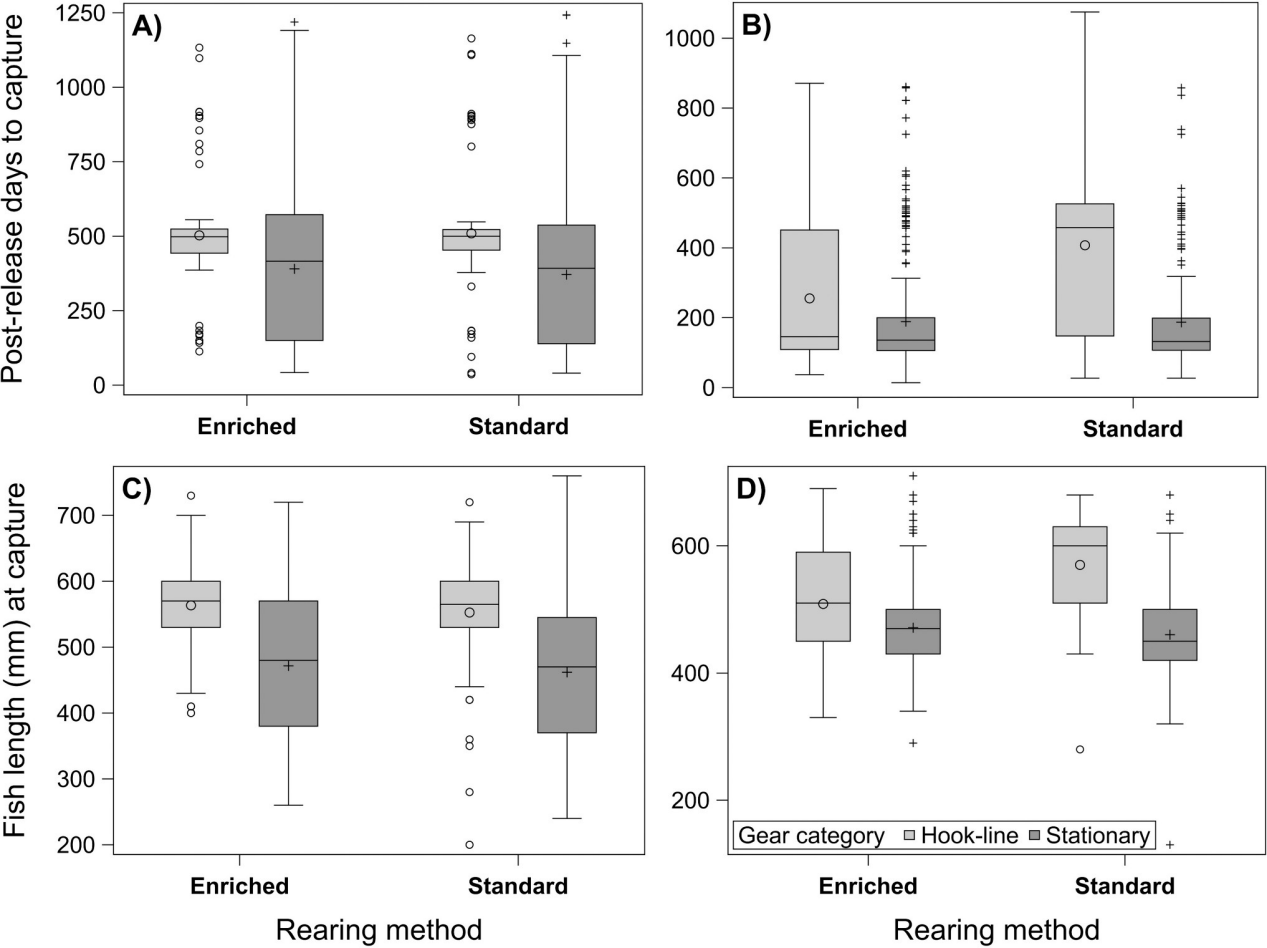
Boxplots illustrating data distribution for capture time and body length of 2-yo (left graphs) and 3-yo salmon in relation to rearing method, with hook-and-line and stationary gear categories separated. The boxes outline the interquartile range (IQR), with median shown as a horizontal line and mean as symbols. Outliers located >1.5 IQR from the box outlines are shown as individual points. Boxplots for capture time and fish length distributions in enriched- and standard-reared salmon released at ages 2 and 3.

Neither the time until the 2-yo fish were caught nor their body length at capture showed a significant difference of means between the rearing methods (Table 5). By contrast, a statistical difference between the rearing methods was found in both response variables in the 3-yo fish, when the significant effect of initial fish TL was corrected for in the model (Table 5): the fish grown in enriched environment were caught earlier and at smaller size, on average, compared to the standard-reared fish. In both age groups, the effect of fishing method (hook and line vs. stationary gears) was similar and significant on both capture rate and fish length. That is, the fish were caught earlier (2-yo: *t*_485_ = 6.72, *p* < 0.001; 3-yo: *t*_587_ = 6.26, *p* < 0.001) and at smaller size (2-yo: *t*_467_ = 9.39, *p* < 0.001; 3-yo: *t*_572_ = 9.45, *p* < 0.001) with stationary gears (Fig 7A–7D). For the fish stocked as 2-yo, the effect of fishing method was similar in standard and enriched groups (non-significant interaction term in both response traits; Table 5; Fig 7A and 7C). Instead, a significant interaction between rearing and fishing method was found in fish stocked as 3-yo (Table 5). The fish reared in enriched environment were captured considerably earlier (*t*_587_ = 7.09, *p* < 0.001) and at smaller size (*t*_572_ = 4.35, *p* < 0.001) with hook and line gears compared to standard fish (Fig 7B and 7D), but there was no such effect in fish captured with stationary gears.

**Table 5:** Results from statistical tests concerning the effects of rearing method, fishing method category, and initial fish length (at tagging; TL) and condition factor^a^ (CF) on time (days post-release) and fish length at capture in 2- and 3-yo salmon. Insignificant interaction and covariate terms were excluded from the final models.

Age group	Trait	Effect	d.f.	*F*-value	*p*-value
**2-yo**	Days until	Rearing method	1, 484	0.36	0.550
	captured	Fishing method	2, 484	23.37	<0.001
		Rearing × Fishing method	2, 482	0.30	0.744
		Initial fish TL	1, 484	11.25	0.001
		Initial fish CF	1, 482	0.61	0.434
	Fish length	Rearing method	1, 467	1.20	0.274
		Fishing method	2, 467	48.76	<0.001
		Rearing × Fishing method	2, 465	0.00	0.995
		Initial fish length	1, 466	0.98	0.322
**3-yo**	Days until	Rearing method	1, 587	9.12	0.003
	captured	Fishing method	2, 587	23.60	<0.001
		Rearing × Fishing method	2, 587	7.43	0.001
		Initial fish TL	1, 587	6.57	0.011
		Initial fish CF	1, 584	1.87	0.172
	Fish length	Rearing method	1, 572	16.84	<0.001
		Fishing method	2, 572	45.53	<0.001
		Rearing × Fishing method	2, 572	14.82	<0.001
		Initial fish TL	1, 571	0.24	0.622

Statistical test results for the effects of rearing method, fishing method category, and initial fish length and condition factor on post-release days and fish length at capture.

^**a**^Effect of covariate fish CF was tested for days until captured.

Irrespective of the release age, the time between release and capture shortened with increasing initial fish length [2-yo: estimate -0.050 (95% CI -0.079– -0.021), 3-yo: -0.001 (-0.002–0.000; Table 5). Initial CF instead showed no significant effect on capture time in either age group, and neither was any of the interactions between covariate (TL or CF) and rearing group or fishing method significant (excluded from the final models). For the fish stocked as 2-yo, the random effect due to release site was negligible for both capture time and fish length at capture (i.e. variance could not be estimated).

In total, 262 tag returns (26.6%) were obtained from the fish that were tested for migration behavior before the release. The likelihood of capture was significantly affected by rearing method (*z* = -2.45, *p* = 0.014; enriched-reared fish being more vulnerable) and PC2 score (estimate -0.188 ± 0.073 SE; *z* = -2.57, *p* = 0.010) but not by PC1 score (0.011 ± 0.075 SE; *z* = 0.15, *p* = 0.885). Thus, earlier time of maximal activity increase was associated with an increased risk of being captured when all fishing methods were combined. This result was independent of the rearing method (non-significant interaction terms between rearing method and PCs). There was no correlation between PCs and the post-release capture time in either of the rearing groups (Pearson’s *r* = -0.105– -0.022, *p* ≥ 0.281).

## Discussion

The present results revealed that enrichment of the hatchery environment with structural complexity and periodical water flow and depth changes can influence the phenotype and performance of landlocked Atlantic salmon in several ways. In comparison with conventionally reared fish, the fish grown in enriched conditions showed better survival during the first year and higher body condition (relative body mass) after 2 and 3 summers of rearing. As a result of better survival, realized rearing density was unintendedly higher in enriched treatment in the first year. This, together with changes in water level and the consequent changes in fish density through the rearing period, possibly contributed to the larger growth variation and higher incidence of dorsal fin damages in comparison with standard rearing. Smolt migration tendency of 3-yo fish was, on average, at the same level between the rearing groups, but the activity distribution was narrower in enriched-reared fish. Rearing method did not influence the experimentally assessed vulnerability to angling, but declining body condition during the previous summer increased the risk of becoming captured. Based on post-stocking tag returns from large lake fisheries, rearing method did not affect the capture rates when the fish were released at the age of 2 years. However, enriched-reared fish had a significantly higher risk to become captured (were caught more frequently with stationary gears) than the standard-reared fish, when the fish were released at the age of 3 years. Moreover, the enriched-reared fish were captured faster than their standard-reared conspecifics with hook and line gears, mainly trolling. Because the experimental angling only revealed a strong vulnerability effect for fish condition, our results suggest that the present rearing methods induced growth and behavior-related differences that indirectly explain differential capture rates in lake fisheries.

### Performance in hatchery rearing

As expected, enriched rearing significantly increased the early survival of fish over standard rearing. Most mortality occurred during the confirmed outbreaks of *Ichthyobodo* sp. parasites and *Flavobacterium* sp. bacteria (first year), causing a survival difference of 21%-units between the rearing methods. Since the water to the hatchery came from a common source, survival differences between the rearing methods supposedly arose due to treatment differences. Further, the observed survival rates were consistent among the replicate tanks within treatments suggesting a marginal role for pure coincidence. Nevertheless, we cannot resolve whether the treatment differences arose from environmental factors or from differences in fish resistance and tolerance. Overall, these results are consistent with previous observations on salmonids [33, 34, 46], and support the idea that progression of certain pathogen- and parasite-induced diseases can be restrained by simple enrichment solutions of the aquaculture environment. It is expected that additional structures provide pathogens with extra surface and hinder the self-cleaning nature of tanks by trapping food and feces [47]. Yet, more heterogeneous environment might also promote beneficial or harmless microbiome both in diversity and quantity and as such inhibit outside host pathogen reproduction particularly in bacteria such as *Flavobacterium* [48, 49]. In any case, disease control by environmental enrichments clearly deserves further studies.

Larger growth variation of fish in enriched conditions in relation to standard-reared group contrasted with our presumption. This can be explained, at least in part, by the higher early survival of the enriched-reared fish because early mortality can be size-selective and thus truncate size variation [50]. However, the observed size heterogeneity within the enriched rearing group, together with fin damages, more obviously resulted from a density-dependent competition for access to shelter [22, 38, 39]. Sudden changes in water level were indeed potential stressors, when altered fish density induced competition among individuals through the rearing period. In general, growth variation may reflect intrinsically differential risk-taking behaviors among individuals so that bold fish with higher motivation to forage grow better than fish with a stronger tendency to hide [51]. In two previous studies, shelter-enrichment was found to negatively affect the growth of Atlantic salmon [22, 26]. Rosengren et al. [22] concluded that decreased growth in enriched-reared fish may be explained by their preference for hiding instead of feeding when shelters are available. Reduced visual field and irregular flow dynamics as such may also diminish the foraging rate in an enriched environment. It remains to be tested, whether a more varying environment, where physical structures change places throughout the experiment, could decrease the chance of fish to establish stable territories.

In captive rearing of salmonids, phenotypic growth variation is typically increased by social hierarchies, where dominant individuals tend to monopolize food resources at the expense of others [52–54]. In this sense, the present findings do not support the assumption that environmental enrichment alleviates hierarchical social structure and reduces aggressive behaviors among individuals [35, 36]. Likewise, fin damages can be used as an indicator of the level of aggressive interactions and chronic stress [22, 36, 39], though they may also result from other causes such as mechanical abrasions against hard substrates [55, 56]. In the present study, the fin erosions were predominant in dorsal fin, and *Flavobacterium* infections were suspected as one (if not the main) causative agent; there were also signs of inflammation in the dorsal fin of many fish. Consequently, the higher incidence of dorsal fin erosions in enriched-reared fish could also arise, to some extent, from their better survival during the first summer, relative to standard-reared fish, reflecting traces of disease outbreaks rather than the effects of enrichment itself. High rearing density or low feed ration has been shown to incur negative effects on fin condition of salmonids [21, 22, 57]. Chronic crowding stress and damaged fins may in turn increase the exposure of fish to pathogens such as water mold (saprolegniosis). However, in this study the survival of fish in enriched environment was not compromised at any stage of rearing in relation to the standard-reared group.

### Smolt migration

Similarly as anadromous salmon, the studied landlocked population undergo physiological, morphological and behavioral changes related to smoltification that prepare the fish for feeding migration [31, 58]. During this transformation, total body and muscle lipid contents decrease, causing a decrease in condition factor. The rearing method affected smolt migration behavior by diverging the temporal distribution of the activity between the groups. Although individual variation in migration patterns was pronounced in both rearing groups, without observable difference in treatment means, the distribution of activity (PC1) was clearly more condensed (peaked) among the fish reared in enriched environment. According to expectations [40, 59], the tendency to migrate was strongly affected by body length and maturity status, longer and immature fish maintaining higher migration activity and attaining the fastest migration speed earlier. Rosengren et al. [22] found that migration success in sea-running Atlantic salmon smolts (age 1-yo) released into the River Imsa, Norway, was strongly positively correlated with fish length regardless of the rearing method (standard vs. enriched and high vs. low rearing density). In another study, Atlantic salmon smolts reared using a similar enrichment method as in the present study showed significantly faster initial migration speed in River Tornionjoki, Finland, and this could have further contributed to their superior survival (2-fold increase) in comparison to the standard-reared fish [24]. In the present study, a more consistent migration activity displayed by the enriched group is, nevertheless, surprising, given their larger size variation (TL: CV% = 8.3%, BW: CV% = 25.6%, *n* = 495 fish) compared to the standard group (6.1 and 11.0%, respectively, *n* = 491). In salmonids, both smolting and maturation processes are linked to early growth rate, and a certain threshold size and adequate energy reserves must be attained in order to adopt certain developmental routes [60, 61]. Migration distance as such, however, may not be tightly dependent on smolt size, but other factors related to rearing environment presumably play a larger role: hatchery-reared salmon smolts are generally bigger but have shorter feeding migration distances than wild smolts [4, 62].

It is presumable that most fish in our study had already smoltified at 2 years of age when the migration tests could not yet be arranged for logistic reasons [31, 63], although it remains unknown whether and to what extent the strength of new smolting was reduced a year later. On the other hand, the degree of smoltification may have also varied more in 2-yo fish than in the studied 3-yo fish. In brown trout (*Salmo trutta*), smolt migration distance at the ages of 2 and 3 years was found to be individually highly repeatable (intraclass correlation coefficient 0.608) [41]. Although the monitoring period in our experiment was fairly short (30 days) and all fish might not have attained their individual peak migration activity, our experiment likely captured relevant biological variation in individual smolt migration tendencies. Yet, a longer duration of monitoring would be needed to fully evaluate the entire migration dynamics of the smolts in this population.

### Vulnerability to fishing

The post-stocking tag-recovery data revealed that the proportion of tag returns was substantially higher for the fish stocked as 3-yo (approx. 30%) than for fish stocked as 2-yo (14%). However, most of the 3-yo stocked fish were caught within six months of the release, and the relative number of fish captured at smaller than the minimum size limit (500 mm) was also much larger for 3-yo (61%) than for 2-yo group (35%). Irrespective of the release age, the captures of undersized fish were mostly attributable to stationary gears (gillnets). The average time between the release and capture was also shorter in fishing with stationary gears than in hook and line fishing, mainly trolling. Yet, in reality the number of undersized fish caught using hook and line gears was probably much larger than what the tag returns indicated, because in most cases such fish can be released quickly back to the water without noting the tag or registering its code.

The number of tag returns from 3-yo fish captured with stationary gears was clearly higher for the enriched (271) than for the standard group (195). Moreover, there was a gear category -dependent difference in capture rates of 3-yo enriched-reared fish, relative to the standard-reared group (when the significant effect of initial fish size was statistically controlled). This was explained by the significantly earlier average capture time of fish in the enriched rearing group by hook and line gears. These differences may result from 1) different behavior (foraging and moving activity), 2) different survival or 3) different spatial distribution of the fish groups.

Different behavior is a plausible explanation for capture rate differences, because high foraging activity likely increases vulnerability to hook and line gears and high moving activity vulnerability to all sorts of stationary gears [64–66]. Rodewald et al. [17] found that enriched rearing improves foraging rate and utilization of natural prey in Atlantic salmon parr compared with fish from a standard rearing environment. In brown trout, high exploration tendency increased an individual’s susceptibility to angling, although high exploration rate during a short-term experiment was promoted by standard rather than enriched rearing [25, 67]. Braithwaite and Salvanes [14] in turn showed in cod (*Gadus morhua*) that more variable rearing conditions (spatial heterogeneity) promoted exploration rate in a novel environment and recovery from a stressful experience in relation to fish reared in a plain environment. Nevertheless, behavioral responses may largely be dependent upon the enrichment type applied and the environment type after release, i.e. river vs. large lake: when Atlantic salmon juveniles received natural prey and were conditioned to threat of predation they displayed two times lower willingness for risk-taking behavior than the fish reared under standard hatchery conditions [68].

Survival differences between the groups could explain differential capture rates, particularly because the angling experiment did not suggest rearing treatment-dependent differences in the individual vulnerability of salmon to angling. There are previous indications that enriched or seminatural rearing methods may increase post-release survival in salmonids [20, 24, 69]. However, the absence of a difference in fish released at the age of 2 years does not support a significant role for survival. Long-term series of stocking experiments on Chinook salmon (*Oncorhynchus tshawytscha*) showed that a variety of environmental enrichments in raceways, including in-stream woody debris structures, overhead covers and underwater feeding, did not consistently increase smolt or smolt-to-adult survival, in comparison with conventionally reared fish [70, 71]. Between-group difference in survival might be considered a less likely explanation for the tag return rate differences in the present study, because the 3-yo fish in both groups were on average large enough to avoid predation mortality by the most abundant gape-limited, relatively small pikeperch or short-term starvation [72, 73]. The 2-yo fish were more vulnerable to predation, and yet there were no differences in their tag return rates between the rearing methods. Furthermore, even though the enriched environment within the hatchery may better prepare the fish to avoid predation by training them to use shelter [74], this characteristic is unlikely beneficial in the pelagic feeding habitat of the salmon. There was no apparent difference in the spatial distribution of fish captures between the rearing methods excluding the third alternative explanation (Fig 6). Thus, while the relative roles of behavior and survival cannot be disentangled with the available data, the observed differences in post-stocking capture rates between the rearing methods suggest that environmental enrichment can influence the capture rates by various fishing gears, but that such effects may be dependent on the release age of the fish. Because of the technical failure during the transportation of the 2-yo fish for release, and the subsequent potentially sublethal effects on the released fish from enriched rearing, the lack of treatment differences should be interpreted with care.

Irrespective of the age at stocking, the time the fish spent in the lake before capture diminished with increasing fish length at release. This is expected when fishing habits or regulations imply positively size-selective fishing, i.e. the faster the fish grow the faster they recruit to fishing. Hyvärinen and Vehanen [75] found a similar positive relationship between the capture rate and the fish length at release in brown trout in Lake Oulujärvi, Finland. A recent study by Vainikka et al. [76] also showed that the capture probability of lake-stocked brown trout was positively associated with both fish length and condition at release. However, the fish captured by hook and line gears exhibited, on average, lower realized post-release growth rate, compared to the fish captured by gillnets. This observation together with the result obtained from the present angling experiment indicates that weak nutritional status of fish (hunger) may increase their vulnerability to fishing with artificial baits. In general, fish body size is the single most important trait that contributes to vulnerability to capture; large fish are more susceptible to fishing than smaller fish due to size selectivity of gears, higher food intake needs, and increased activity [67]. In the present study, the 3-yo fish grown in enriched environment had higher average body size and condition than the standard-reared fish at stocking (difference of means 42 g in BW and 0.10 in CF; S1 Table), suggesting that these attributes contributed to the more frequent captures of the first group (i.e., larger fish with better condition probably grew faster to a vulnerable size range where captures by gillnets are possible). However, while capture probability in experimental angling was independent on fish length, and declining body condition appeared to predict high vulnerability, the catch data from the lake supported rather an opposite pattern: the time between stocking and capture was unaffected by initial fish condition, regardless of the fishing method, whereas the impact of fish length (and rearing method) was significant. Increased vulnerability of 3-yo fish to lake fisheries was also predicted by early achievement of maximal migration speed in artificial stream channels (PC2). This suggests that fishing might also induce selection on smolt migration traits by selecting against traits that physiologically explain early smoltification; high growth and body condition probably play a significant role also in this respect. Nevertheless, neither the likelihood nor timing of capture was associated with total migration movement (PC1), suggesting that mobility during smolt migration and in lacustrine feeding area represent different activity traits.

## Conclusions

The results of this study have significant implications for the management of landlocked salmon fisheries. The undersized (3-yo) fish were vulnerable to a strong fishing pressure by size-selective and lethal gillnets, presumably increasing instant or nearly instant post-release mortality. The prevalent use of gillnets with small mesh sizes in Lake Höytiäinen is mainly due to unrestrained recreational and commercial fishing for pikeperch (*Sander lucioperca*) [72]. Finally, feasible improvements to commercial rearing practices are needed to increase the efficiency of stocking programs. The present study on landlocked Atlantic salmon demonstrated that rearing method can have long-term effects on the performance of fish in a natural lake environment with realized differences in fisheries capture rates. Based on the representative tag recovery statistics, we conclude that the applied environmental enrichment during hatchery rearing induced plastic phenotypic responses that resulted in increased tag recoveries soon after the stocking. However, this effect was observed only in fish released at 3 years of age, suggesting the stronger effect for the hatchery environment the longer the fish are reared in hatchery conditions. During hatchery rearing, environmental enrichment generated favorable effects on the early survival of juveniles, likely through decreased mortality to fish parasites and diseases. This is an important aspect regarding the preservation of genetic variation in captive breeding programs. Yet, considering increased incidence of fin damages at later developmental stages relative to standard rearing, the applied enrichment design may not appear ideal in every respect within the whole smolt production period. Overall, the poor management of lake fisheries led to the capture of the stocked fish long before their growth potential was realized. In this respect, relatively better stocking results could be obtained by improving the fisheries management than by altering rearing methods only. The environmental enrichment during hatchery rearing did not improve the capacity of fish to avoid fishing mortality at young age and small size. Further studies on the effects of rearing methods on both pre- and post-release performance should incorporate careful behavioral observations and alternative arrangements, where the relative importance of different components (e.g. shelter vs. changes in water level and currents or exposure to predator scent) could be resolved.

## Supporting information

S1 Table Number of 3-yo fish measured, means and variations in total length (TL), body weight (BW) and condition factor (CF), and the percentage of matured males in standard and enriched rearing groups. Means and variations in total length, body weight and condition factor, and the percentage of matured males in standard and enriched rearing groups of 3-year-old salmon.
(DOCX)

S2 Table Number of 3-yo salmon (% in brackets) in standard and enriched rearing groups belonging to different fin erosion categories. Number of enriched- and standard-reared 3-year-old salmon in different fin erosion categories.
(DOCX)

S1 Fig Variation of water temperature during the study period (23 Apr 2009–30 May 2012) at Kainuu Fisheries Research Station. Variation of water temperature during the study period at Kainuu Fisheries Research Station.
(DOCX)

S1 Data (ZIP)
