# Supplementary material for: The effects of environmental enrichment on hatchery-performance, smolt migration and capture rates in landlocked Atlantic salmon
Source: PLoS One. 2021 Dec 2;16(12):e0260944. doi: 10.1371/journal.pone.0260944 (PMC8638868; doi:10.1371/journal.pone.0260944)
Supplement: S1 Table — Means and variations in total length, body weight and condition factor, and the percentage of matured males in standard and enriched rearing groups of 3-year-old salmon. (DOCX) [file pone.0260944.s001.docx]

**S1 Table. Number of 3-yo fish measured, means and variations in total length (TL), body weight (BW) and condition factor (CF), and the percentage of matured males in standard and enriched rearing groups.**

| **Rearing** | ***n*** | **TL (mm)** | | **BW (g)** | | **CF** | **Maturity-%** |
| --- | --- | --- | --- | --- | --- | --- | --- |
|  |  | mean ± SD | CV% | mean ± SD | CV% | mean ± SD |  |
| **Standard** | 995 | 267 ± 17 | 6.4 | 165.2 ± 31.9 | 19.3 | 0.78 ± 0.08 | 13.5 |
| **Enriched** | 993 | 276 ± 23 | 8.3 | 206.9 ± 51.8 | 25.0 | 0.88 ± 0.10 | 16.9 |
