# Supplementary material for: The effects of environmental enrichment on hatchery-performance, smolt migration and capture rates in landlocked Atlantic salmon
Source: PLoS One. 2021 Dec 2;16(12):e0260944. doi: 10.1371/journal.pone.0260944 (PMC8638868; doi:10.1371/journal.pone.0260944)
Supplement: S2 Table — Number of enriched- and standard-reared 3-year-old salmon in different fin erosion categories. (DOCX) [file pone.0260944.s002.docx]

**S2 Table.** **Number of 3-yo salmon (% in brackets) in standard and enriched rearing groups belonging to different fin erosion categories.**

|  |  | **Fin erosion score^a^** | | | | |
| --- | --- | --- | --- | --- | --- | --- |
| **Fin** | **Rearing** | **0** | **1** | **2** | **3** | **4** |
| **Dorsal** | Standard | 143 (14.3) | 68 (6.8) | 234 (23.5) | 289 (29.0) | 263 (26.4) |
|  | Enriched | 44 (4.4) | 40 (4.0) | 171 (17.2) | 437 (43.9) | 303 (30.5) |
| **Right** | Standard | 722 (72.4) | 186 (18.7) | 72 (7.2) | 16 (1.6) | 1 (0.1) |
| **pectoral** | Enriched | 733 (73.7) | 178 (17.9) | 62 (6.2) | 14 (1.4) | 8 (0.8) |
| **Left** | Standard | 679 (68.1) | 176 (17.7) | 105 (10.5) | 26 (2.6) | 11 (1.1) |
| **pectoral** | Enriched | 827 (83.1) | 123 (12.4) | 41 (4.1) | 3 (0.3) | 1 (0.1) |
| **Right** | Standard | 948 (95.1) | 31 (3.1) | 13 (1.3) | 4 (0.4) | 1 (0.1) |
| **pelvic** | Enriched | 890 (89.4) | 62 (6.2) | 24 (2.4) | 11 (1.1) | 8 (0.8) |
| **Left** | Standard | 927 (93.1) | 29 (2.9) | 26 (2.6) | 6 (0.6) | 8 (0.8) |
| **pelvic** | Enriched | 895 (89.9) | 62 (6.2) | 19 (1.9) | 9 (0.9) | 10 (1.0) |

^a^0 = pristine, 1 = erosion <25%, 2 = erosion approx. 50%, 3 = erosion approx. 75%, 4 = full erosion.
