# Supplementary material for: The effects of environmental enrichment on hatchery-performance, smolt migration and capture rates in landlocked Atlantic salmon
Source: PLoS One. 2021 Dec 2;16(12):e0260944. doi: 10.1371/journal.pone.0260944 (PMC8638868; doi:10.1371/journal.pone.0260944)

**S3 Figure.** **Variation of water temperature during the study period (23 Apr 2009 – 30 May 2012) at Kainuu Fisheries Research Station.**


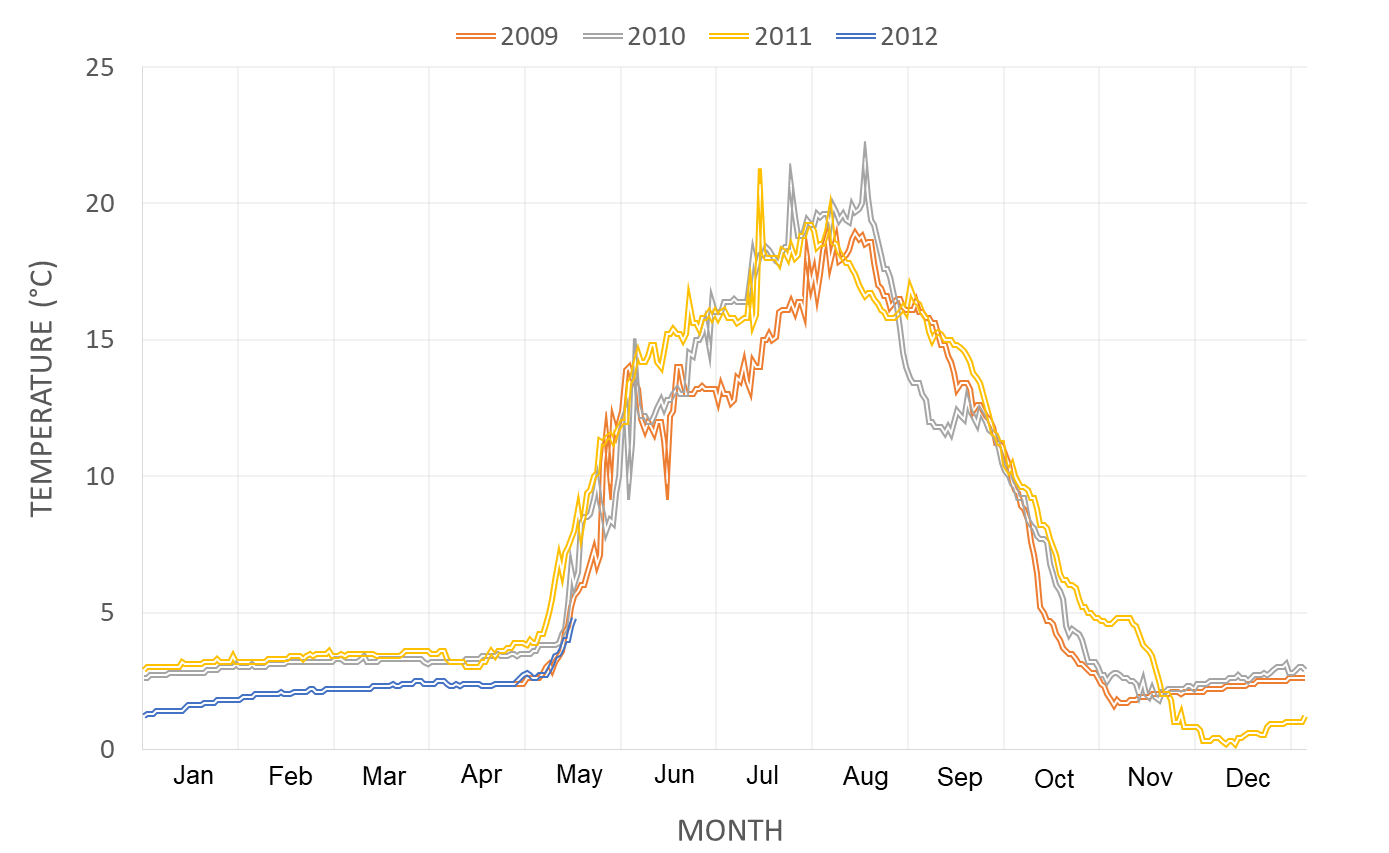

Supplement: S1 Fig — Variation of water temperature during the study period at Kainuu Fisheries Research Station. (DOCX) [file pone.0260944.s003.docx]
